# Synthesis of polybrominated benzimidazole and benzotriazole derivatives containing a tetrazole ring and their cytotoxic activity

**DOI:** 10.1007/s00706-016-1785-8

**Published:** 2016-06-14

**Authors:** Edyta Łukowska-Chojnacka, Patrycja Wińska, Monika Wielechowska, Maria Bretner

**Affiliations:** Faculty of Chemistry, Institute of Biotechnology, Warsaw University of Technology, Noakowskiego St. 3, 00-664 Warsaw, Poland

**Keywords:** Antitumor agents, Azole, Benzimidazole, Heterocycles, Tetrazole, Total synthesis

## Abstract

**Abstract:**

A series of new benzimidazole and benzotriazole derivatives containing a tetrazole moiety was synthesized by *N*-alkylation of 5-aryltetrazole with 4,5,6,7-tetrabromo-1-(3-chloropropyl)-1*H*-benzimidazole and 4,5,6,7-tetrabromo-2-(3-chloropropyl)-2*H*-benzotriazole. The reaction was regioselective and mostly 2,5-disubstituted tetrazole derivatives were obtained. The effect of all synthesized compounds on human recombinant casein kinase 2alpha subunit (rhCK2α) and cytotoxicity against human T-cell lymphoblast (CCRF-CEM) and breast adenocarcinoma (MCF-7) cell lines were evaluated. The results have shown that many of the synthesized compounds exhibit significant cytotoxicity at micromolar concentration.

**Graphical abstract:**

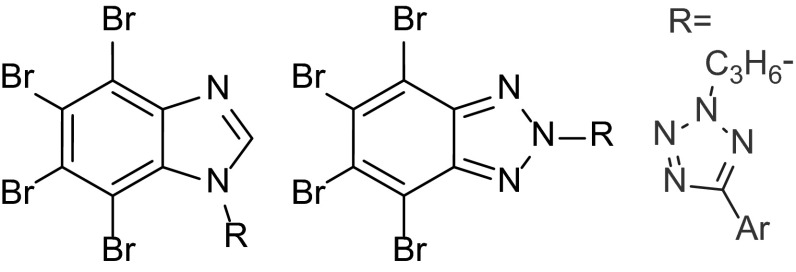

## Introduction

Azole derivatives have been reported to possess diverse biological activity and are widely used in a medicinal chemistry [1–3]. Nowadays, many research groups focus on designing and synthesis of compounds containing two or more azoles, in search for obtaining more active compounds. Many structures containing indole, benzotriazole, or benzimidazole linked with tetrazolyl substituent have been obtained and characterized. It was shown that the attachment of a tetrazolyl substituent to indole derivatives resulted in an increase of both; antibacterial activity against Gram-positive bacteria (*Bacillus subtilis*, *Streptococcus lactis*) and Gram-negative bacteria (*Escherichia coli*, *Pseudomonas aeruginosa*), and antifungal activity against *Candida albicans*, *Aspergillus niger*, and *Penicillium sp*. [4]. Furthermore, it is known, that certain indolyltetrazole derivatives exhibit significant anticancer activity against human liver carcinoma cell line (HepG2) [5]. The incorporation of the tetrazole moiety into benzotriazole and benzimidazole derivatives resulted in the improvement of biological action of the compound. Structures containing both benzotriazole and tetrazole moiety in addition to antibacterial and antifungal properties exhibit significant, anticonvulsant, anti-inflammatory, and anti-nociceptive activity [6, 7]. Compound 2-[2-(5-phenyl-1*H*-tetrazol-1-yl)phenyl]-1*H*-benzimidazole exhibits significant antibacterial (against *Staphylococcus aureus* and *Escherichia coli*) and antifungal (against *C. albicans* and *A.niger*) activity comparable with ciprofloxacin and fluconazole, respectively [8]. Similar properties are also described for 2-(α-*p*-substituted phenyl-α-benzimidazolo)methyl-1,2,3,4-tetrazole [9] and other derivatives [10, 11]. Furthermore, there are known benzimidazole-tetrazole derivatives, which show anti-hypertensive activity compared with losartan and telmisartan [12–14]. There are also obtained benzimidazole derivatives bearing a tetrazole ring, that reveal moderate anti-asthmatic, and anti-diabetic activity as well as antioxidant and antitumor activity against mice Dalton’s lymphoma ascites (DLA), human breast adenocarcinoma (MCF-7), and human colon adenocarcinoma (HCT) cell lines [11, 15–18].

Taking into consideration the biological importance of benzimidazole, benzotriazole, and tetrazole derivatives and fact that the fusion of various tetrazoles to azoles generally leads to an increase of the biological activity, we decided to synthesize a new group of compounds containing benzimidazole/benzotriazole and tetrazole moieties and evaluate their cytotoxic effect on two human cancer cell lines: T-cell lymphoblast (CCRF-CEM) and breast adenocarcinoma (MCF-7). Knowing that 4,5,6,7-tetrabromo-1*H*-benzotriazole (TBBt) and 4,5,6,7-tetrabromo-1*H*-benzimidazole (TBBi) derivatives are inhibitors of casein kinase CK2 [19, 20], which is significantly increased in many tumor cells [21, 22], these derivatives were chosen for further modifications.

## Results and discussion

### Chemistry

Synthesis of the intermediate and target compounds was performed according to the Scheme 1. The starting polybrominated benzimidazole **1** and benzotriazole **2** were obtained according to the described in literature methods [23, 24]. Substrates **3** and **4** were obtained by *N*-alkylation of 4,5,6,7-tetrabromo-1*H*-benzimidazole (**1**) or 4,5,6,7-tetrabromo-2*H*-benzotriazole (**2**) with 1-bromo-3-chloropropane. The alkylation was performed in the presence of KOH as a base in acetonitrile at 60 °C [25]. The new azoles **6a**–**6d**, **7a**–**7d**, and **8a**–**8d** were synthesized by *N*-alkylation of 5-aryltetrazoles **5a**–**5d** with 4,5,6,7-tetrabromo-1-(3-chloropropyl)-1*H*-benzimidazole (**3**) and 4,5,6,7-tetrabromo-2-(3-chloropropyl)-2*H*-benzotriazole (**4**). The 5-aryltetrazoles **5a**–**5d** were obtained from commercially available nitriles, NaN_3_, and NH_4_Cl in DMF according to the described method [26].

**Figure:**
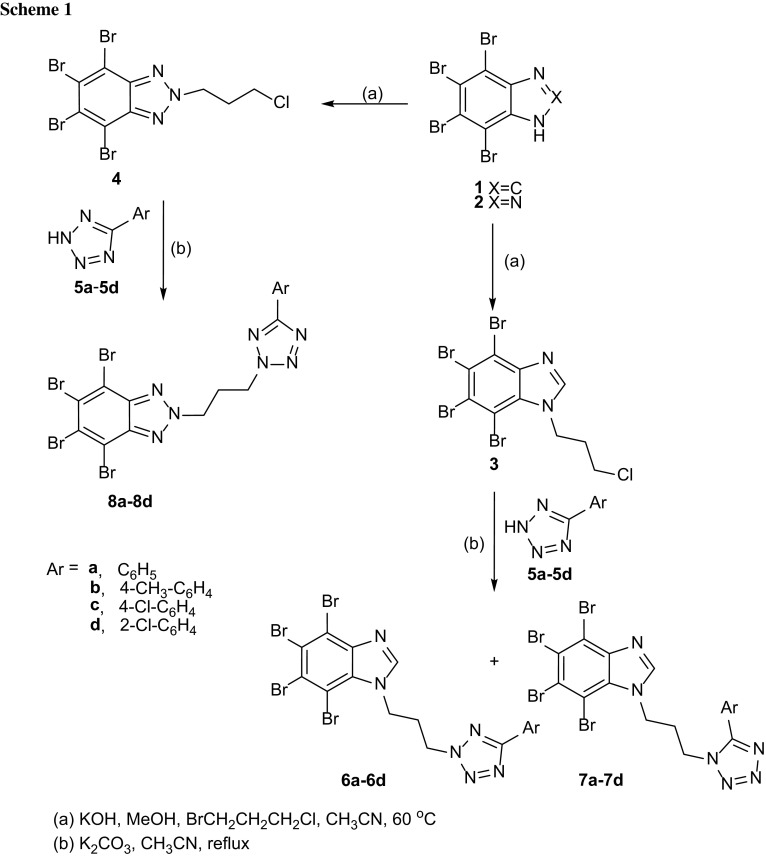

First attempt of synthesis of compounds **6a** and **7a** was performed in *n*-butanol in the presence of K_2_CO_3_ and KI at 90 °C, but in these conditions products were not formed. In other approach only K_2_CO_3_ and CH_3_CN were used. The reaction was performed at reflux and the progress was monitored by thin-layer chromatography (Scheme 1). Products **6a** and **7a** were obtained with total yield 86 %. The ratio of 2,5-disubstituted isomer **6a** to 1,5-disubstituted isomer **7a** equaled 7.42 to 1, and showed the regioselectivity of the reaction. In this manner *N*-alkylation of other aryltetrazole derivatives **5b**–**5d** was performed. In all cases reactions were regioselective and formation of 2,5-disubstituted tetrazoles was preferred. Additionally, the weight ratio of 2,5-isomer **6a**–**6d** to 1,5-isomer **7a**–**7d** depended on aryl substituent in the tetrazole ring and range from 13.5/1 to 2.8/1. The times, yields, and isomers ratios are summarized in Table 1. All isomeric products **6a**–**6d** and **7a**–**7d** were separated and purified by column chromatography on silica gel. Their structures were elucidated on the basis of ^1^H NMR, ^13^C NMR, IR, and HRMS spectral data.

**Table 1:** The reaction times and yields of the compounds **6a**–**6d**, **7a**–**7d**, and **8a**–**8d**

Comp	Ar	Time/h	Yield/%	Isomers ratio (**6**/**7**)
**6a**+**7a**	C_6_H_5_–	48	86	7.42/1
**6b**+**7b**	4-CH_3_–C_6_H_4_–	48	89	7.41/1
**6c**+**7c**	4-Cl–C_6_H_4_–	48	80	13.5/1
**6d**+**7d**	2-Cl–C_6_H_4_–	48	78	2.8/1
**8a**	C_6_H_5_–	48	57	1/0
**8b**	4-CH_3_–C_6_H_4_–	48	46	1/0
**8c**	4-Cl–C_6_H_4_–	48	54	1/0
**8d**	2-Cl–C_6_H_4_–	240	63	1/0

The same procedure was applied for the synthesis of 4,5,6,7-tetrabromo-2*H*-benzotriazole derivatives **8a**–**8d**. The progress of the reaction was monitored by TLC using chloroform:cyclohexane (9:1 v/v) as an eluent. Products were purified three times by column chromatography. There is no doubt, that repeated purification contributed to lower yields of the reactions (Table 1). In this case only 2,5-disubstituted tetrazoles were obtained, what is consistent with our previous studies [27].

### Cytotoxicity assay

The effect of synthesized compounds on viability of human T lymphoblast leukemia (CCRF-CEM) and human breast adenocarcinoma (MCF-7) cell lines was evaluated using MTT assay [28]. The cytotoxicity of 4,5,6,7-tetrabromo-1*H*-benzimidazole (**1**) and its derivatives **3**, **6a**–**6d**, and **7a**–**7d** was determined after 24 h and 48 h treatment at 10, 25, 50, and 100 μM concentration of each compound (Tables 2, 3).

**Table 2:** Effect of 4,5,6,7-tetrabromo-1*H*-benzimidazole (**1**) and its derivatives **3**, **6a**–**6d**, and **7a**–**7d** on viability of CCRF-CEM cells

Comp	CCRF-CEM cell viability % ± SD							
	Incubation time 24 h				Incubation time 48 h			
	10 μM	25 μM	50 μM	100 μM	10 μM	25 μM	50 μM	100 μM
**1**	112 ± 10	51 ± 2	15 ± 5	1 ± 0	90 ± 9	23 ± 2	3 ± 1	0 ± 0
**3**	97 ± 7	67 ± 10	4 ± 1	1 ± 1	103 ± 5	69 ± 3	3 ± 2	0 ± 0
**6a**	106 ± 6	73 ± 10	4 ± 1	6 ± 1	105 ± 14	64 ± 1	0 ± 0	1 ± 0
**6b**	108 ± 8	92 ± 0.5	18 ± 3	9 ± 2	113 ± 5	77 ± 0	6 ± 1	1 ± 1
**6c**	103 ± 11	91 ± 6	45 ± 9	31 ± 4	124 ± 6	102 ± 1	31 ± 3	15 ± 2
**6d**	102 ± 8	96 ± 2	68 ± 10	52 ± 1	104 ± 3	89 ± 1	42 ± 3	31 ± 4
**7a**	67 ± 9	4 ± 1	2 ± 1	2 ± 0	57 ± 7	0 ± 0	0 ± 0	0 ± 0
**7b**	82 ± 5	7 ± 1	6 ± 2	6 ± 3	68 ± 7	9 ± 1	1 ± 0	0 ± 0
**7c**	90 ± 12	19 ± 3	7 ± 2	6 ± 2	66 ± 7	12 ± 3	0 ± 0	0 ± 0
**7d**	65 ± 5	1 ± 0.6	2 ± 1	2 ± 2	51 ± 12	1 ± 0	0 ± 0	0 ± 0

Data are presented as mean ± SD in three repeated experiments

**Table 3:** Effect of 4,5,6,7-tetrabromo-1*H*-benzimidazole (**1**) and its derivatives **3**, **6a**–**6d**, and **7a**–**7d** on viability of MCF-7 cells

Comp	MCF-7 cell viability % ± SD							
	Incubation time 24 h				Incubation time 48 h			
	10 μM	25 μM	50 μM	100 μM	10 μM	25 μM	50 μM	100 μM
**1**	89 ± 2	71 ± 3	56 ± 0	44 ± 2	88 ± 4	57 ± 4	37 ± 5	25 ± 2
**3**	97 ± 4	88 ± 2	45 ± 1	6 ± 2	96 ± 5	66 ± 1	31 ± 10	4 ± 2
**6a**	104 ± 1	110 ± 1	47 ± 3	40 ± 5	103 ± 2	70 ± 4	31 ± 6	19 ± 3
**6b**	103 ± 4	83 ± 3	21 ± 1	7 ± 1	98 ± 4	67 ± 2	19 ± 5	2 ± 0
**6c**	98 ± 4	59 ± 3	19 ± 2	5 ± 2	97 ± 8	76 ± 0	13 ± 7	3 ± 1
**6d**	100 ± 4	80 ± 3	25 ± 5	9 ± 3	98 ± 3	67 ± 4	25 ± 2	19 ± 6
**7a**	ND	ND	ND	ND	64 ± 2	22 ± 4	16 ± 2	10 ± 1
**7b**	88 ± 5	77 ± 6	75 ± 0	52 ± 4	87 ± 5	28 ± 1	29 ± 3	28 ± 3
**7c**	98 ± 3	62 ± 7	68 ± 1	63 ± 0	87 ± 3	29 ± 0	21 ± 6	20 ± 1
**7d**	89 ± 1	69 ± 9	68 ± 3	61 ± 5	91 ± 6	45 ± 1	25 ± 4	16 ± 1

Data are presented as mean ± SD in three repeated experiments

*ND* not determined

In the case of the leukemia cell line (CCRF-CEM), the highest cytotoxicity exhibited compounds **7a** and **7d**, that at the 25 μM concentration caused a complete (**7a**) or almost complete (**7d**) loss of cell viability after 48 h treatment. Worth attention are also compounds **7b**, **7c**, that effectively inhibited cell growth at the 50 μM concentration. As can be seen from the results presented in the Table 2 the cytotoxic activity of investigated compounds depended on their structure. All 1,5-disubsituted tetrazoles **7a**–**7d** appeared more cytotoxic than their 2,5-disubstituted isomers **6a**–**6d**. It has to be pointed out, that compounds **7a**–**7d** exhibited higher cytotoxicity than TBBi (**1**) and its 3-chloropropyl derivative **3**.

Human breast adenocarcinoma cell line (MCF-7) reveals higher resistance for all tested compounds as compared with leukemia cell line (Table 3). All benzimidazole derivatives exhibited lower cytotoxicity against MCF-7 than it was observed for CCRF-CEM cell line, and higher compound concentration and/or prolongation of treatment time to 48 h were necessary to observe significant decrease of cell viability. The most active compounds were **7a**–**7d** (25 μM) and **6b**–**6d** (50 μM). Moreover, comparing the cytotoxicity of TBBI (**1**) and 4,5,6,7-tetrabromo-1-(3-chloropropyl)-1*H*-benzimidazole (**2**), with the effect of these compounds, it appeared that synthesized derivatives **7a**–**7d** and **6b**–**6d** reduced the viability of MCF-7 cells more effectively.

Similar experiments with 4,5,6,7-tetrabromo-2*H*-benzotriazole (**2**) and its derivatives **4** and **8a**–**8d** were performed. The results are summarized in Tables 4 and 5. In contrary to above-described compounds, the benzotriazole derivatives **8a**–**8d** exhibited lower cytotoxicity against CCRF-CEM then against MCF-7 cancer cell lines.

**Table 4:** Effect of 4,5,6,7-tetrabromo-2*H*-benzotriazole (**2**) and its derivatives **4** and **8a**–**8d** on viability of CCRF-CEM cells

Comp	CCRF-CEM cell viability % ± SD					
	Incubation time 24 h			Incubation time 48 h		
	25 μM	50 μM	100 μM	25 μM	50 μM	100 μM
**2**	94.3 ± 1.2	62.1 ± 4.2	25.4 ± 3.5	78.9 ± 3.0	54.6 ± 1.8	7.5 ± 0.6
**4**	81.9 ± 4.0	85.7 ± 5.1	90.6 ± 2.7	82.1 ± 8.1	78.2 ± 0.6	76.6 ± 5.6
**8a**	81.5 ± 4.6	49.7 ± 4.7	34.7 ± 4.8	73.8 ± 4.7	41.0 ± 1.8	35.5 ± 0.9
**8b**	89.1 ± 3.7	70.9 ± 0.8	67.1 ± 3.7	93.8 ± 0.6	74.7 ± 3.4	70.1 ± 3.1
**8c**	46.9 ± 2.2	40.7 ± 3.7	31.0 ± 6.0	48.6 ± 3.8	39.4 ± 1.7	12.9 ± 1.8
**8d**	88.7 ± 0.5	88.1 ± 6.3	94.3 ± 8.0	94.0 ± 5.5	76.9 ± 4.0	70.9 ± 1.9

Data are presented as mean ± SD in three repeated experiments

**Table 5:** Effect of 4,5,6,7-tetrabromo-2*H*-benzotriazole (**2**) and its derivatives **4** and **8a**–**8d** on viability of MCF-7 cells

Comp	MCF-7 cell viability % ± SD					
	Incubation time 24 h			Incubation time 48 h		
	25 μM	50 μM	100 μM	25 μM	50 μM	100 μM
**2**	102.1 ± 5.6	95.1 ± 2.4	77.9 ± 3.9	93.9 ± 1.4	85.6 ± 1.6	51.1 ± 0.8
**4**	74.1 ± 2.7	66.8 ± 1.9	59.2 ± 4.7	53.4 ± 1.0	55.0 ± 3.6	60.3 ± 5.0
**8a**	67.0 ± 2.2	47.0 ± 1.6	25.3 ± 2.0	60.6 ± 2.9	30.7 ± 1.8	3.7 ± 0.7
**8b**	57.4 ± 1.7	49.7 ± 3.3	33.8 ± 2.3	40.0 ± 2	35.3 ± 2.3	19.5 ± 3.4
**8c**	38.2 ± 1.3	23.0 ± 2.6	5.1 ± 2.4	13.5 ± 2.0	2.8 ± 0.4	1.3 ± 0.4
**8d**	80.3 ± 3.0	70.8 ± 1.4	52.0 ± 3.3	64.8 ± 2.4	58.1 ± 2.4	32.7 ± 3.0

Data are presented as mean ± SD in three repeated experiments

For the CCRF-CEM cell line the highest cytotoxicity was observed for compounds **8a** and **8c**, which at 50 µM concentration, regardless of incubation time, reduced the cell viability to about 40 % (Table 4).

For the MCF-7 the most significant inhibitory effect was observed for compounds **8a**–**8c**, which showed cytotoxicity at all investigated concentrations in time-dependent manner (Table 5). These compounds were also more cytotoxic then TBBt (**2**) and 4,5,6,7-tetrabromo-2-(3-chloropropyl)-2*H*-benzotriazole (**4**).

It can be concluded that the viability of tested human cancer cell lines was reduced by all synthesized compounds in the concentration- and time-dependent manner. It is also evident, that these close structurally related molecules displayed remarkable differences in cytotoxicity. Benzimidazole derivatives containing tetrazole ring appeared more cytotoxic against both cell lines then their benzotriazole analogs. It has to be pointed out, that many of the synthesized azoles (**7a**–**7d**, **6a**–**6d**, **8a**–**8c**) showed higher cytotoxicity than basic compounds TBBi **1** or TBBt **2** and their corresponding chloropropyl derivatives **3** and **4**, what confirms, that the aryltetrazolyl substituent can increase the biological activity.

To explain the molecular mechanism of action of the synthesized tetrazole derivatives, their effect on the activity of human recombinant casein kinase 2α subunit (rhCK2α) was estimated. Unexpectedly, all obtained derivatives did not inhibit kinase activity at 1–20 micromolar concentrations, what indicates that this enzyme is not a target for these compounds. It is known that TBBt and TBBi and their derivatives affect other kinases and proteins not dependent on ATP, i.e. quinone reductase [29], or interacting with ATP, i.e., heat shock proteins HSP90B or HSP7C [30] therefore further investigation of inhibitory activity and protein interactions of obtained compounds are needed.

## Conclusion

A series of twelve new azoles was synthesized by the reaction of 4,5,6,7-tetrabromo-1-(3-chloropropyl)-1*H*-benzimidazole (**3**) and 4,5,6,7-tetrabromo-2-(3-chloropropyl)-2*H*-benzotriazole (**4**) with various 5-aryltetrazoles **5a–5d**. Products were obtained in relatively short time and with satisfactory yields. In all reactions, formation of 2,5-disubstituted tetrazoles was preferred. For all compounds, the influence on human casein kinase CK2α subunit and cytotoxic activity against human CCRF-CEM and MCF-7 cancer cell lines were evaluated. All synthesized benzimidazole derivatives exhibited higher cytotoxicity against CCRF-CEM cells than against MCF-7 cells, and in the case of benzotriazole derivatives opposite trend was observed. According to our expectations, the introduction of aryltetrazole moiety into the alkyl side chain of the benzimidazole or benzotriazole resulted in higher cytotoxicity as compared to the nonsubstituted TBBi (**1**) and TBBt (**2**), and its analogs **3** and **4**. It has been proved that the cytotoxic effect of synthesized compounds is not caused by the interaction with rhCK2α subunit.

## Experimental

All reagents, solvents, and chemicals were purchased from POCH (Gliwice, Poland), Merck (Darmstadt, Germany), and Sigma-Aldrich (Munich, Germany) and were at analytical grade. Dimethyl sulfoxide (DMSO), molecular biology grade used as a solvent for all stocks of the chemical agents was obtained from Roth (Karlsruhe-Rheinhafen, Germany). The reactions were monitored by TLC aluminum plates with silica gel Kieselgel 60 F_254_ (Merck, Darmstadt, Germany, 0.2 mm thickness film) using UV light as visualizing agent. Column chromatography was performed using Kieselgel 60 (Merck, 0.040–0.063 mm). Melting points were determined in open glass capillary tubes. IR spectra were taken on a Specord M80 instrument (Carl Zeiss, Jena, Germany). HRMS spectra were recorded on a Micro-mass ESI Q-ToF Premier instrument (Micromass UK Limited, Manchester UK). ^1^H (400 MHz) and ^13^C NMR (100 MHz) spectra were recorded on a Mercury 400 MHz spectrometer (Varian, California, USA) in CDCl_3_ solution; chemical shifts (*δ*) are reported in ppm; coupling constants (*J*) are given in hertz (Hz).

### *General procedure for N*-*alkylation of 5*-*aryltetrazoles****5a***–***5d****with 4,5,6,7*-*tetrabromo*-*1*-*(3*-*chloropropyl)*-*1H*-*benzimidazole (****3****) and 4,5,6,7*-*tetrabromo*-*2*-*(3*-*chloropropyl)*-*2H*-*benzotriazole (****4****)*

A mixture of appropriate 5-aryltetrazole (**5a**–**5d**, 1 mmol) and 0.18 g K_2_CO_3_ (1 mmol) in 30 cm^3^ acetonitrile was stirred and heated to reflux. After 30 min 0.26 g 4,5,6,7-tetrabromo-1-(3-chloropropyl)-1*H*-benzimidazole (**3**, 0.5 mmol) or 0.25 g 4,5,6,7-tetrabromo-2-(3-chloropropyl)-2*H*-benzotriazole (**4**, 0.5 mmol) was added and the reaction mixture was stirred at reflux. The progress of the reaction was monitored by TLC with chloroform–methanol 9:1 v/v (in the case of benzimidazole derivatives) and chloroform:cyclohexane 9:1 v/v (in the case of benzotriazole derivatives) as the eluent. After completion of the reaction the mixture was cooled in an ice bath and 20 cm^3^ of chloroform was added. The inorganic solids were filtered off and the solvent was evaporated. Products **6a**–**6d** and **7a**–**7d** were separated and purified using column chromatography on silica gel with a chloroform–methanol (9:1 v/v) as the eluent. Products **8a**–**8d** were purified three times on column chromatography on silica gel with a chloroform-hexane (9:1 v/v) and cyclohexane-ethyl acetate (10:3 v/v) as the eluent. The combined fractions of each product were concentrated and analyzed.

#### *4,5,6,7*-*Tetrabromo*-*1*-*[3*-*(5*-*phenyl*-*2H*-*tetrazol*-*2*-*yl)propyl]*-*1H*-*benzimidazole* (**6a**, C_17_H_12_Br_4_N_6_)

Yield: 0.2353 g (76 %); colorless crystals; m.p.: 174–176 °C; ^1^H NMR (400 MHz, CDCl_3_): *δ* = 8.16 (brs, 1H, CH), 8.12–8.14 (m, 2H, Ar), 7.50–7.51 (m, 3H, Ar), 4.69–4.72 (m, 2H, CH_2_N), 4.60–4.62 (m, 2H, *CH*_*2*_NCH), 2.64–2.67 (m, 2H, CH_2_*CH*_*2*_CH_2_) ppm; ^13^C NMR (100 MHz, CDCl_3_): *δ* = 165.49, 147.04, 144.14, 130.93, 130.65, 129.01, 126.81, 126.79, 124.13, 122.36, 117.82, 105.96, 49.24, 43.75, 31.44 ppm; IR (nujol): $\bar{v}$  = 1525, 1493, 1452, 1401, 1353, 1197, 1153, 1102, 1070, 1051, 1022, 940, 901 cm^−1^; HRMS (ESI-TOF): *m*/*z* = 620.8308 ([M + 1]^+^).

#### *4,5,6,7*-*Tetrabromo*-*1*-*[3*-*[5*-*(4*-*methylphenyl)*-*2H*-*tetrazol*-*2*-*yl]propyl]*-*1H*-*benzimidazole* (**6b**, C_18_H_14_Br_4_N_6_)

Yield 0.2475 g (78 %); colorless crystals; m.p.: 126–128 °C; ^1^H NMR (400 MHz, CDCl_3_): *δ* = 8.23 (brs, 1H, CH), 8.00–8.02 (m, 2H, Ar), 7.30–7.32 (m, 2H, Ar), 4.68–4.71 (m, 2H, CH_2_N), 4.60–4.64 (m, 2H, *CH*_*2*_NCH), 2.64–2.67 (m, 2H, CH_2_*CH*_*2*_CH_2_), 2.43 (s, 3H, CH_3_) ppm; ^13^C NMR (100 MHz, CDCl_3_): *δ* = 165.59, 147.07, 140.90, 130.92, 129.70, 126.70, 124.22, 124.03, 122.46, 117.72, 106.03, 49.18, 43.83, 31.44, 21.53 ppm; IR (nujol): $\bar{v}$ = 1617, 1579, 1528, 1449, 1410, 1340, 1200, 1165, 1095, 1070, 1041, 1025, 984, 946 cm^−1^; HRMS (ESI-TOF): *m*/*z* = 634.8370 ([M + 1]^+^).

#### *4,5,6,7*-*Tetrabromo*-*1*-*[3*-*[5*-*(4*-*chlorophenyl)*-*2H*-*tetrazol*-*2*-*yl]propyl]*-*1H*-*benzimidazole* (**6c**, C_17_H_11_Br_4_ClN_6_)

Yield 0.243 g (74 %); colorless crystals; m.p.: 155–157 °C; ^1^H NMR (400 MHz, CDCl_3_): *δ* = 8.12 (brs, 1H, CH), 8.05–8.07 (m, 2H, Ar), 7.47–7.49 (m, 2H, Ar), 4.69–4.72 (m, 2H, CH_2_N), 4.60–4.64 (m, 2H, *CH*_*2*_NCH), 2.64–2.70 (m, 2H, CH_2_*CH*_*2*_CH_2_) ppm; ^13^C NMR (100 MHz, CDCl_3_); *δ* = 164.62, 146.95, 144.13, 136.74, 130.91, 129.33, 128.06, 125.31, 124.18, 122.41, 117.86, 105.94, 49.39, 43,80, 31.34 ppm; IR (nujol): $\bar{v}$ = 1608, 1576, 1550, 1493, 1442, 1417, 1340, 1204, 1168, 1092, 1051, 1016, 997, 978, 943 cm^−1^; HRMS (ESI-TOF): *m*/*z* = 654.7910 ([M + 1]^+^).

#### *4,5,6,7*-*Tetrabromo*-*1*-*[3*-*[5*-*(2*-*chlorophenyl)*-*2H*-*tetrazol*-*2*-*yl]propyl]*-*1H*-*benzimidazole* (**6d**, C_17_H_11_Br_4_ClN_6_)

Yield 0.1877 g (57 %); colorless crystals; m.p.: 149–151 °C; ^1^H NMR (400 MHz, CDCl_3_): *δ* = 8.23 (s, 1H, CH), 7.99–8.01 (m, 1H, Ar), 7.55–7.57 (m, 1H, Ar), 7.42–7.45 (m, 2H, Ar), 4.72–4.75 (m, 2H, CH_2_N), 4.65–4.68 (m, 2H, *CH*_*2*_NCH), 2.64–2.70 (m, 2H, CH_2_*CH*_*2*_CH_2_) ppm; ^13^C NMR (100 MHz, CDCl_3_): *δ* = 163.63, 147.18, 144.21, 132.92, 131.39, 131.25, 130.96, 127.08, 125.92, 124.10, 122.36, 117.83, 105.98, 49.18, 43.63, 31.56 ppm; IR (nujol): $\bar{v}$ = 1598, 1569, 1525, 1496, 1449, 1410, 1366, 1340, 1197, 1168, 1130, 1092, 1064, 1041, 1006, 980, 940 cm^−1^; HRMS (ESI-TOF): *m*/*z* = 654.7910 ([M + 1]^+^).

#### *4,5,6,7*-*Tetrabromo*-*1*-*[3*-*(5*-*phenyl*-*1H*-*tetrazol*-*1*-*yl)propyl]*-*1H*-*benzimidazole* (**7a**, C_17_H_12_Br_4_N_6_)

Yield 0.0317 g (10 %); colorless crystals; m.p.: 116–118 °C; ^1^H NMR (400 MHz, CDCl_3_): *δ* = 8.04 (s, 1H, CH), 7.55–7.60 (m, 3H, Ar), 7.49–7.53 (m, 2H, Ar), 4.62–4.66 (m, 2H, CH_2_N), 4.42–4.46 (m, 2H, *CH*_*2*_NCH), 2.46–2.53 (m, 2H, CH_2_*CH*_*2*_CH_2_) ppm; ^13^C NMR (100 MHz, CDCl_3_): *δ* = 154.45, 146.74, 144.14, 131.62, 130.83, 129.44, 1286.52, 124.19, 123.26, 122.43, 117.87, 105.86, 44.19, 43.76, 31.85 ppm; IR (nujol): $\bar{v}$ = 1576, 1541, 1493, 1468, 1449, 1404, 1379, 1353, 1197, 1178, 1130, 1105, 1073, 1038, 1019, 946 cm^−1^; HRMS (ESI-TOF): *m*/*z* = 620.8217 ([M + 1]^+^).

#### *4,5,6,7*-*Tetrabromo*-*1*-*[3*-*[5*-*(4*-*methylphenyl)*-*1H*-*tetrazol*-*1*-*yl]propyl]*-*1H*-*benzimidazole* (**7b**, C_18_H_14_Br_4_N_6_)

Yield 0.0334 g (10 %); colorless crystals; m.p.: 192–193 °C; ^1^H NMR (400 MHz, CDCl_3_): *δ* = 8.04 (s, 1H, CH), 7.46–7.48 (m, 2H, Ar), 7.29–7.31 (m, 2H, Ar), 4.62–4.65 (m, 2H, CH_2_N), 4.42–4.45 (m, 2H, *CH*_*2*_NCH), 2.49–2.51 (m, 2H, CH_2_*CH*_*2*_CH_2_), 2.44 (s, 3H, CH_3_) ppm; ^13^C NMR (100 MHz, CDCl_3_): *δ* = 154.47, 146.76, 144.13, 142.24, 130.86, 130.12, 128.37, 124.16, 122.39, 120.25, 117.85, 105.80, 44.21, 43.78, 31.80, 21.57 ppm; IR (nujol): $\bar{v}$ = 1620, 1582, 1554, 1525, 1490, 1480, 1452, 1407, 1379, 1353, 1185, 1130, 1108, 1038, 949, 905 cm^−1^; HRMS (ESI-TOF): *m/z* = 634.8370 ([M + 1]^+^).

#### *4,5,6,7*-*Tetrabromo*-*1*-*[3*-*[5*-*(4*-*chlorophenyl)*-*1H*-*tetrazol*-*1*-*yl]propyl]*-*1H*-*benzimidazole* (**7c**, C_17_H_11_Br_4_ClN_6_)

Yield 0.018 g (6 %); colorless crystals; m.p.: 214–215 °C; ^1^H NMR (400 MHz, CDCl_3_): *δ* = 8.08 (s, 1H, CH), 7.50–7.57 (m, 4H, Ar), 4.66–4.69 (m, 2H, CH_2_N), 4.41–4.45 (m, 2H, *CH*_*2*_NCH), 2.50–2.53 (m, 2H, CH_2_*CH*_*2*_CH_2_) ppm; ^13^C NMR (100 MHz, CDCl_3_): *δ* = 153.58, 146.70, 144.13, 138.29, 130.79, 129.89, 129.82, 124.27, 122.56, 121.63, 117.94, 105.80, 44.29, 43.69, 31.77 ppm; IR (nujol): $\bar{v}$ = 1598, 1449, 1404, 1356, 1185, 1121, 1089, 1013, 975, 953 cm^−1^; HRMS (ESI-TOF): *m*/*z* = 654.8099 ([M + 1]^+^).

#### *4,5,6,7*-*Tetrabromo*-*1*-*[3*-*[5*-*(2*-*chlorophenyl)*-*1H*-*tetrazol*-*1*-*yl]propyl]*-*1H*-*benzimidazole* (**7d**, C_17_H_11_Br_4_ClN_6_)

Yield 0.066 g (20 %); colorless crystals; m.p.: 83–86 °C; ^1^H NMR (400 MHz, CDCl_3_): *δ* = 8.01 (s, 1H, CH), 7.50–7.53 (m, 2H, Ar), 7.40–7.42 (m, 2H, Ar), 4.61–4.65 (m, 2H, CH_2_N), 4.20–4.24 (m, 2H, *CH*_*2*_NCH), 2.41–2.45 (m, 2H, CH_2_*CH*_*2*_CH_2_) ppm; ^13^C NMR (100 MHz, CDCl_3_): *δ* = 152.68, 146.74, 144.13, 133.48, 133.01, 131.66, 130.79, 130.28, 127.58, 124.19, 123.14, 122.44, 117.86, 105.89, 44.07, 43.89, 31.53 ppm; IR (nujol): $\bar{v}$ = 1601, 1525, 1487, 1449, 1401, 1344, 1165, 1134, 1111, 1080, 1032, 971 cm^−1^; HRMS (ESI-TOF): *m/z* = 654.7910 ([M + 1]^+^).

#### *2*-*[3*-*(5*-*Phenyl*-*2H*-*tetrazol*-*2*-*yl)propyl]*-*2H*-*benzotriazole* (**8a**, C_16_H_11_Br_4_N_7_)

Yield 0.173 g (57 %); colorless crystals; m.p.: 185–189 °C (dec.); ^1^H NMR (400 MHz, DMSO-*d*_*6*_): *δ* = 7.67–7.69 (m, 2H, Ar), 7.41–7.44 (m, 3H, Ar), 4.99–5.20 (m, 2H, CH_2_N), 4.92–4.95 (m, 2H, CH_2_N), 2.80–2.86 (m, 2H, CH_2_) ppm; ^13^C NMR (100 MHz, DMSO-*d*_*6*_): *δ* = 163.38, 142.25, 131.22, 130.29, 127.18, 126.08, 125.59, 125.39, 118.04, 113.32, 55.10, 50.96, 28.20 ppm; IR (nujol): $\bar{v}$ = 1579, 1525, 1512, 1452, 1426, 1178, 1124, 1083, 1045, 975 cm^−1^; HRMS (ESI-TOF): *m/z* = 6217295 ([M + 1]^+^).

#### *2*-*[3*-*[5*-*(4*-*Methylphenyl)*-*2H*-*tetrazol*-*2*-*yl]propyl]*-*2H*-*benzotriazole* (**8b**, C_17_H_13_Br_4_N_7_)

Yield 0.145 g (46 %); colorless crystals; m.p.: 177–178 °C; ^1^H NMR (400 MHz, DMSO-*d*_*6*_): *δ* = 7.47–7.49 (m, 2H, Ar), 7.17–7.19 (m, 2H, Ar), 4.98–5.01 (m, 2H, CH_2_N), 4.91–4.94 (m, 2H, CH_2_N), 2.80–2.85 (m, 2H, CH_2_), 2.35 (s, 3H, CH_3_) ppm; ^13^C NMR (100 MHz, DMSO-*d*_*6*_): *δ* = 163.28, 142.14, 139.92, 129.26, 125.34, 123.16, 113.22, 55.25, 51.06, 28.22, 21.18 ppm; IR (nujol): $\bar{v}$ = 1449, 1430, 1178, 1130, 1067, 1045, 975 cm^−1^; HRMS (ESI-TOF): *m/z* = 635.7364 ([M + 1]^+^).

#### *2*-*[3*-*[5*-*(4*-*Chlorophenyl)*-*2H*-*tetrazol*-*2*-*yl]propyl]*-*2H*-*benzotriazole* (**8c**, C_16_H_10_Br_4_ClN_7_)

Yield 0.173 g (54 %); colorless crystals; m.p.: 193–195 °C; ^1^H NMR (400 MHz, CDCl_3_): *δ* = 7.89–7.91 (m, 2H, Ar), 7.41–7.45 (m, 2H, Ar), 4.93–4.96 (m, 2H, CH_2_N), 4.85–4.88 (m, 2H, CH_2_N), 2.95–3.02 (m, 2H, CH_2_) ppm; ^13^C NMR (100 MHz, CDCl_3_): *δ* = 164.15, 143.12, 136.53, 129.12, 127.75, 126.72, 125.09, 113.61, 54.73, 50.63, 28.80 ppm; IR (nujol): $\bar{v}$ = 1636, 1601, 1560, 1531, 1442, 1356, 1176, 1165, 1127, 1096, 1076, 1038, 1013, 981 cm^−1^; HRMS (ESI-TOF): *m*/*z* = 655.7704 ([M + 1]^+^).

#### *2*-*[3*-*[5*-*(2*-*Chlorophenyl)*-*2H*-*tetrazol*-*2*-*yl]propyl]*-*2H*-*benzotriazole* (**8d**, C_16_H_10_Br_4_ClN_7_)

Yield 0.2 g (63 %); colorless crystals; m.p.: 153–154 °C; ^1^H NMR (400 MHz, DMSO-*d*_*6*_): *δ* = 7.69–7.71 (m, 1H, Ar), 7.41–7.57 (m, 3H, Ar), 4.99–5.20 (m, 2H, CH_2_N), 4.95–4.99 (m, 2H, CH_2_N), 2.78–2.86 (m, 2H, CH_2_) ppm; ^13^C NMR (100 MHz, DMSO-*d*_*6*_): *δ* = 161.82, 142.34, 131.68, 131.41, 130.69, 130.62, 127.28, 125.43, 125.21, 113.45, 54.83, 50.81, 28.19 ppm; IR (nujol): $\bar{v}$ = 1608, 1573, 1528, 1445, 1426, 1200, 1172, 1130, 1070, 1045, 981 cm^−1^; HRMS (ESI-TOF): *m*/*z* = 655.6667 ([M + 1]^+^).

### Cell culture and treatment

MCF-7 adherent cells (human breast adenocarcinoma line) were cultured in DMEM medium with high glucose (Gibco), supplemented with 10 % fetal bovine serum (Gibco), 2 mM l-glutamine, antibiotics (100 U/cm^3^ penicillin, 100 µg/cm^3^ streptomycin) and 10 µg/cm^3^ of human recombinant insulin. CCRF-CEM cell suspension (human T lymphoblast cell line) was cultured in RPMI 1640 medium (Gibco) supplemented with 10 % fetal bovine serum and antibiotics (100 U/cm^3^ penicillin, 100 µg/cm^3^ streptomycin). Cells were grown in 25 cm^2^ culture flasks (Sarstedt), in a humidified atmosphere of CO_2_/air (5/95 %) at 37 °C.

### MTT cytotoxicity assay

MCF-7 cells were collected by trypsinization (0.25 % trypsin–EDTA solution, Sigma-Aldrich) and seeded into 96-well microplates at density 1.5–3 × 10^4^ cells/well. At 70 % of confluency (18 h after plating) cells were treated with various concentrations of investigated compounds dissolved in DMSO (0.5 %) or DMSO (0.5 %) alone (control). CCRF-CEM cells were seeded at 2–3 × 10^4^ cells/well and treated with compounds. MTT stock solution (Sigma-Aldrich) was added to each well to a final concentration of 0.5 mg/cm^3^. After 4 h incubation at 37 °C, formazan crystals were dissolved in DMSO (200 mm^3^) (37 °C, 10 min incubation), and Sorensen’s glycine buffer (0.1 M glycine, 0.1 M NaCl, pH 10.5) was added to each well (25 mm^3^/well). Optical densities were measured at 570 nm using BioTek microplate reader. All measurements were carried out in triplicates and the results are expressed as percentage of cell viability relative to control (cells in 0.5 % DMSO). At such conditions, IC_50_ for antitumor antibiotic doxorubicin was 1.4 µM for both MCF-7 and CCRF-CEM lines.

### CK2α assay

CK2α was expressed and purified according to an earlier published procedure [28]. Isotopic assay was used to determine CK2α activity with synthetic peptide substrate (RRRDDDSDDD) from Biaffin GmbH & Co KG, Germany. Experiments were carried out at 30 °C for 20 min in the presence of the increasing concentrations of inhibitors.
